# Identifying hormones and other perioperative risk factors for postoperative delirium after endoscope‐assisted transsphenoidal pituitary adenoma resection: A retrospective, matched cohort study

**DOI:** 10.1002/brb3.3041

**Published:** 2023-05-03

**Authors:** Jin Liu, Jinyu Qian, Xia Wang, Jie Lin, Sunyan Yang, Rong Hu, Jishu Xian, Hua Feng, Yujie Chen, Binbin Tan

**Affiliations:** ^1^ Neurosurgical Intensive Care Unit, Department of Neurosurgery, Southwest Hospital Third Military Medical University (Army Medical University) Chongqing China; ^2^ State Key Laboratory of Trauma, Burn and Combined Injury, Southwest Hospital Third Military Medical University (Army Medical University) Chongqing China; ^3^ Chongqing Brain Science Collaborative Innovation Center Chongqing China; ^4^ Chongqing Clinical Research Center for Neurosurgery, Southwest Hospital Third Military Medical University (Army Medical University) Chongqing China; ^5^ Chongqing Key Laboratory of Precision Neuromedicine and Neuroregenaration, Southwest Hospital Third Military Medical University (Army Medical University) Chongqing China; ^6^ School of Nursing Guizhou University of Traditional Chinese Medicine Guiyang Guizhou Province China

**Keywords:** corticotropin‐releasing hormone, endoscope‐assisted transsphenoidal surgery, insulin‐like growth factor 1, perioperative risk factor, pituitary adenoma, postoperative delirium

## Abstract

**Objective:**

As a complex and acute brain dysfunction, if postoperative delirium (POD) occurs in the postoperative period, it will lead to a prolonged length of stay in the critical care unit, with increased hospitalization costs and higher mortality. A few case reports inspired us to pay close attention to pituitary tumor‐associated delirium. We hypothesized that the changes in hormone levels after pituitary tumor resection might be associated with POD occurrence.

**Methods:**

Retrospective analysis was performed on data from a single‐center cohort study conducted at Southwest Hospital between January 2018 and May 2022. A total of 360 patients with pituitary tumors who underwent endoscope‐assisted transsphenoidal pituitary tumor resection were divided into two groups at a 1:3 ratio, with 36 patients in the POD group and 108 patients in the non‐POD group matched by propensity score, age, sex, and tumor size. Basic characteristics, pituitary adenoma features, endocrine levels and other biochemical indicators, and Confusion Assessment Method for the Intensive Care Unit (CAM‐ICU) for postoperative delirium were documented for further analysis.

**Results:**

Lower insulin‐like growth factor‐1 (IGF‐1, *p* = .024) and corticotropin‐releasing hormone (CRH, *p* = .005) levels were closely associated with postoperative delirium and with high levels of blood glucose (GLU, *p* = .023) after surgery. Subsequent analysis indicated that serum potassium (OR: 0.311, 95% CI 0.103–0.935), sodium (OR: 0.991, 95% CI 0.983–1.000), CRH (OR: 0.964, 95% CI 0.936–0.994), and GLU (OR: 1.654, 95% CI 1.137–2.406) levels in the perioperative period were independent risk factors for delirium.

**Conclusions:**

Our study indicated that lower serum CRH, potassium, sodium, and GLU levels may be associated with the occurrence of POD after endoscopic‐assisted transsphenoidal surgery. These data provide preliminary evidence for the management of POD in pituitary adenoma patients after surgery. Further studies are needed to identify pharmacological and nonpharmacological multicomponent treatment strategies.

## Graphical abstract

We investigated the potential perioperative risk factors for postoperative delirium in pituitary adenoma patients treated by endoscope‐assisted transsphenoidal surgery. We found that lower IGF‐1 and CRH levels were tightly associated with postdelirium and high levels of blood glucose (GLU) after surgery. Logistic regression analysis indicated that serum potassium (OR 0.311, 95% CI 0.103‐0.935), serum sodium levels (OR 0.991, 95% CI 0.983‐1.000), CRH levels (OR 0.964, 95% CI 0.936‐0.994), and blood glucose (OR 1.654, 95% CI 1.137‐2.406) in the perioperative period were independent risk factors for delirium.

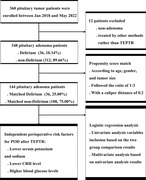

## 1 INTRODUCTION

It has been reported that pituitary adenoma is the second most prevalent primary tumor in the brain parenchyma. In accordance with the progress of neuroendoscopy technology, endoscope‐assisted transsphenoidal surgery for pituitary tumors and other basicranial tumors has led to more radical excision with fewer complications. A recent meta‐analysis showed that the most frequently reported postoperative complications included cerebrospinal fluid leakage (12.8%), diabetes insipidus (12.8%), postoperative hypopituitarism (9.2%), and secondary hematoma (8.5%) (Stefanidis et al., 2022). However, after resection of either functional pituitary adenomas or nonfunctional tumors, endocrine complications are still one of the important factors for patients in neurosurgical intensive care units (NICUs) during the early postoperative period (Keandoungchun et al., 2021). This hormonal status might lead to complex neurological complications, including postoperative delirium, in the NICU (Cruz‐Flores, 2021), which has been reported to be tightly associated with worse cognitive outcomes, including short‐term and long‐term cognitive decline after surgery (Goldberg et al., 2020; Huang et al., 2021).

As a complex and acute brain dysfunction, postoperative delirium is characterized by acute and fluctuating changes in cognition and consciousness. Once delirium appears in the postoperative period, it will lead to a prolonged length of stay in the NICU, increased hospitalization costs and higher mortality; therefore, it is very important to recognize and explore the risk factors for postoperative delirium in patients. To date, only a few case reports have inspired us to pay close attention to pituitary tumor‐associated delirium (Burne et al., 2021; Li et al., 2017; Weng et al., 2008). Even idiopathic pituitary insufficiency could present as prolonged delirium and a “terrible dream” (McAulay‐Powell & Friedman, 2013). Based on these clues, we hypothesized that the changes in hormone levels after pituitary tumor resection might be associated with postoperative delirium occurrence. We enrolled cohorts of patients who underwent endoscope‐assisted transsphenoidal pituitary adenoma resection to analyze the potential perioperative risk factors for postoperative delirium in our NICU ward.

## 2 METHODS

### 2.1 Enrolled patients

We retrospectively enrolled all patients with pituitary tumors admitted to the Department of Neurosurgery, Southwest Hospital between January 2018 and May 2022. The present study was approved by the Ethics Committee of Southwest Hospital of Army Medical University (No. (B)KY2021035) and registered in the Chinese Clinical Trial Registry (http://www.chictr.org.cn/enIndex.aspx, Trial No. ChiCTR2100053607, Registration Date: November 25, 2021). Informed consent to participate was waived due to the observational nature of the study. This study was conducted in accordance with the ethical guidelines of the Helsinki Declaration and reported in accordance with the Strengthening the Reporting of Observational Studies in Epidemiology (STROBE) checklist.

**FIGURE 1:**
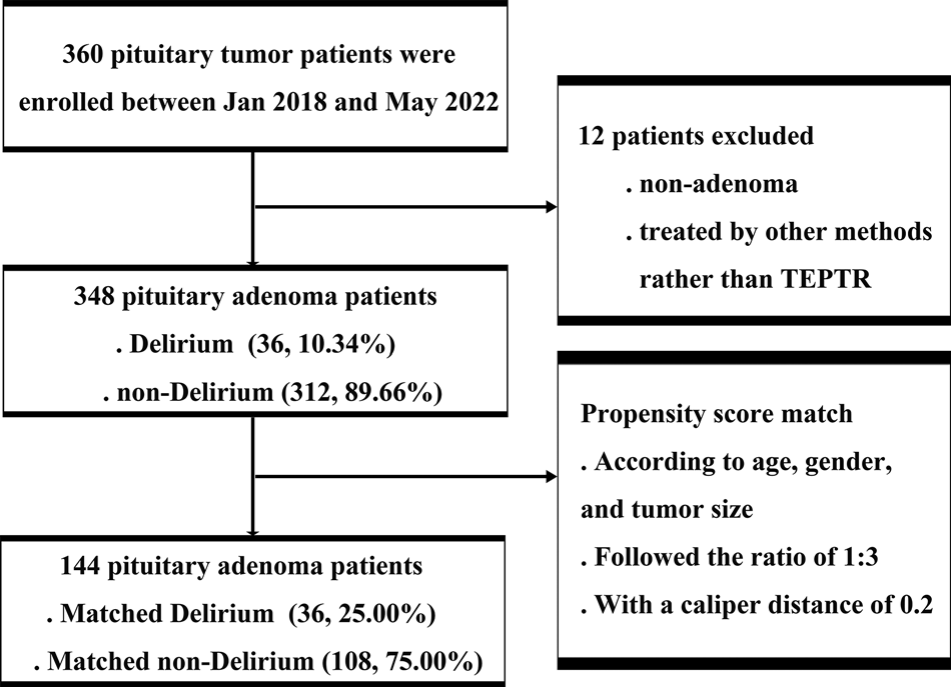
Flow diagram of patient inclusion. Propensity scores were calculated to match the pituitary patients with or without delirium to reduce confounding factors, including age, sex, and tumor size. TEPTR: Trans‐nasal endoscopic pituitary tumor resection.

We selected 360 patients with pituitary tumors who were diagnosed and confirmed by preoperative MRI and endocrinological evaluations and underwent endoscope‐assisted transsphenoidal pituitary tumor resection in our department. Patients with possible undiagnosed dementia or cognitive impairment according to the anamnesis and physical examination record in their medical history were not enrolled. Twelve enrolled patients were excluded due to nonpituitary adenoma confirmed by postoperative biopsy or because of treatment other than endoscope‐assisted transsphenoidal pituitary adenoma resection, and the relevant data of 348 eligible patients with pituitary adenoma were obtained. The flow diagram of the enrolled cohort is illustrated in Figure 1.

Detailed inclusion criteria: (1) age ≥ 18 years old, no sex limitation; (2) pituitary adenoma confirmed by preoperative magnetic resonance imaging (MRI), endocrinology and postoperative pathology and treated by endoscope‐assisted transsphenoidal pituitary tumor resection; (3) GCS score ≥12 on admission; (4) no history of intracranial surgery; and (5) stay in the NICU > 24 h.

Exclusion criteria: (1) previously diagnosed with Alzheimer's disease or other cognitive impairments; (2) communication difficulties, such as speech impairment or language barrier; (3) schizophrenia history or routine use of antipsychiatry drugs; (5) unable to complete delirium assessment due to coma or other reasons; (6) autoimmune diseases, glucocorticoid therapy or use of other hormone drugs; (7) preoperative upper respiratory infection; and (8) patients with unstable vital signs or hemodynamics after surgery.

### 2.2 Delirium diagnosis

The diagnostic criteria for postoperative delirium according to The Diagnostic and Statistical Manual of Mental Disorders, published by the American Psychiatric Association, are as follows: (1) altered mental status represented by a change from baseline or fluctuations in the previous 24 h; (2) inattention; (3) altered consciousness; and (4) disorganized thinking. Postoperative delirium can be diagnosed if the patient has both (1) and (2), plus (3) or (4) (von Hofen‐Hohloch et al., 2020).

After the enrolled patients arrived to the NICU after surgery and were fully resuscitated, two blinded researchers trained in using the Confusion Assessment Method of the Intensive Care Unit (CAM‐ICU) Training Manual performed the delirium evaluation by using the CAM‐ICU Worksheet and Flowsheet (Mandarin Version) (Wang et al., 2013). The patients were routinely evaluated twice a day at 9:00 am and 21:00 pm and additionally screened if the patients exhibited signs of altered mental status. After the patients were transferred out of the NICU, nurses performed the same assessment.

### 2.3 Cohort matching

The CAM‐ICU delirium evaluation showed that 36 patients were positive and 312 patients were negative for delirium. To manage the common characteristics and variates of enrolled patients, we performed propensity score matching for age, sex, and tumor size using a 1:3 ratio. Then, the cohort was divided into two groups according to the presence or absence of postoperative delirium, with 36 patients in the postoperative delirium group (POD group) and 108 patients in the non‐POD group. The flow diagram of cohort matching is also illustrated in Figure 1.

### 2.4 Data extraction

We retrieved the characteristics and clinical data of those matched cohorts via medical records, categorized as (1) basic characteristics, including age, sex, length of stay, operation duration; (2) pituitary adenoma features, including pituitary adenoma classification and size, invasive or not; (3) endocrine levels, including thyroid‐stimulating hormone (TSH), thyroxin (T4), insulin‐like growth factor‐1 (IGF‐1), growth hormone (GH), blood prolactin (PRL), follicle‐stimulating hormone (FSH), estradiol (E2), testosterone, progesterone, adrenocorticotropic hormone (ACTH), and plasma cortisol; (4) other biochemical indicators, including blood sodium, blood potassium, blood glucose, blood chlorine, albumin; and (5) CAM‐ICU for postoperative delirium, intensive care delirium screening checklist (ICDSC) and Richmond agitation‐sedation scale (RASS) for severity of delirium.

### 2.5 Statistical analysis

The 1:3 propensity score match was performed by R version R 4.2.1 (R Foundation for Statistical Computing, Vienna, Austria) on the enrolled subjects via the nearest‐neighbor method without replacement, and the caliper value was set as 0.2 (Figure S1). The data were subsequently analyzed with IBM SPSS Statistics version 24.0 (IBM Corp., Armonk, NY, USA) and diagramed with GraphPad Prism 7.04 (GraphPad Software, USA). Categorical variables were compared with chi‐squared or continuity correction or Fisher's exact test. Continuous data are expressed as the mean ± SD, while discrete data are presented as the median ± IQR. The two independent variables were compared with Student's *t* test or the Mann‒Whitney *U* test. Regression analysis dominantly considered the variables with significant differences in two‐group comparisons. *p* < .05 was regarded as statistically significant, and SMD < 0.1 was considered to indicate that the difference between two groups could be ignored.

## 3 RESULTS

### 3.1 Patient characteristics

The difference in tumor size between the delirium and nondelirium groups could not be ignored before propensity score matching (PSM), and the standard mean difference (SMD) was 0.249. However, there was an obvious decrease in the SMD value and no significant differences in age, female sex, or tumor size between the two matched groups (SMD = 0.076, 0.019, 0.063, Table 1 and Figure S1). In addition, after PSM, the comparisons of baseline data, including adenoma classification, hospital days, and operation times, were not significantly different (*p* = .604, 0.190, 0.729, Table 2).

**TABLE 1:** Propensity score matching according to age, sex and tumor size in pituitary adenoma patients.

Variable		Crude data			PSM data		
	Delirium		SMD	Delirium			SMD
	Yes	No		Yes		No	
Patients, *n* (%)	36 (10.34)	312 (89.66)		36 (25.00)		108 (75.00)	
Demographic							
Age, mean (SD)	49.31 (10.18)	48.20 (12.15)	0.099	49.31 (10.18)		50.11 (11.06)	0.076
Female, *n* (%)	20 (55.6)	162 (51.9)	0.073	16 (44.00)		64 (44.00)	0.019
Tumor size, *n*			0.249				0.063
Microadenoma	0 (0.0)	7 (2.2)		0 (0.0)		0 (0.0)	
Macroadenoma	26 (72.2)	237 (76.0)		26 (72.2)		81 (75.0)	
Giant adenoma	10 (27.8)	68 (21.8)		10 (27.8)		27 (25.0)	

PSM, propensity score matching; SMD, standard mean difference; SD, standard difference.

**TABLE 2:** Clinical data of pituitary adenoma patients with or without delirium.

Variable	Overall	Occurrence of delirium		
		Yes	No	*p* Value
Adenoma classification, *n* (%)				.604
Prolactinoma	9 (6.3)	2 (5.6)	7 (6.5)	
GH adenomas	13 9.0)	2 (5.6)	11 (10.2)	
ACTH adenoma	8 (5.6)	4 (11.1)	4 (3.7)	
Gonadotropin adenoma	20 (13.9)	6 (16.7)	14 (13.0)	
Polysecretory adenoma	3 (2.1)	0	3 (2.8)	
Endocrine‐inactive adenoma	75 (52.1)	18 (50.0)	57 (52.8)	
Invasive pituitary adenoma	3 (2.1)	1 (2.8)	2 (1.9)	
Thyrotroph adenoma	6 (4.2)	2 (5.6)	4 (3.7)	
Adrenal corticosteroid adenoma	2 (1.4)	1 (2.8)	1 (0.9)	
Other	5 (3.5)	0	5 (4.6)	
Hospital days	21.38 (10.11)	24.08 (15.64)	20.48 (7.30)	.190
Operation time	2.69 (1.16)	2.64 (1.09)	2.72 (1.19)	.729

### 3.2 Prognosis and characteristics of postoperative patients with delirium

**FIGURE 2:**
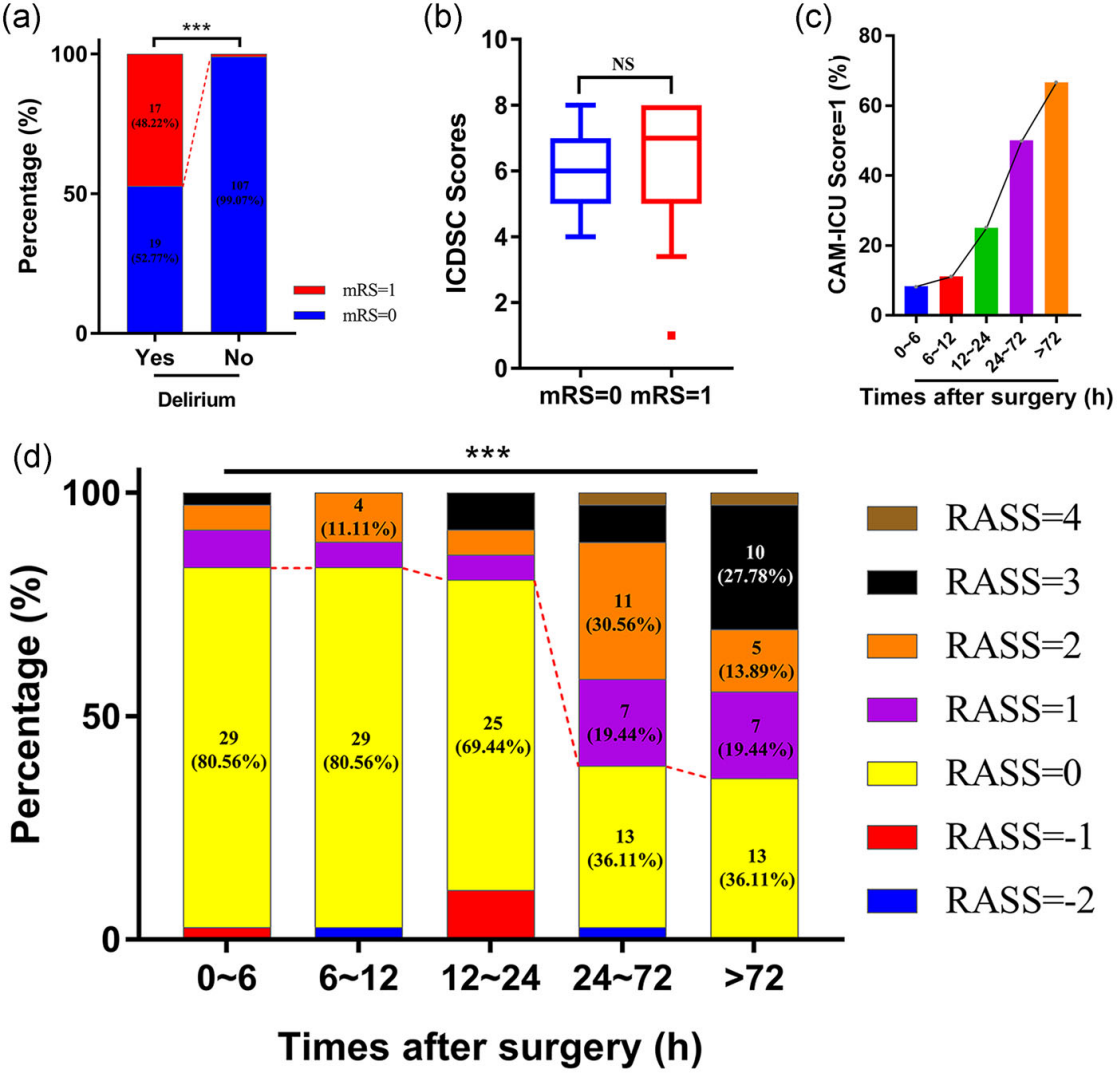
Prognosis and characteristics of postoperation patients with delirium. (A) The postoperative patients with delirium had a larger percentage of poor prognosis (mRS = 1) at discharge than those without delirium. ***χ^2^ = 48.762, *p* < .0001. (B) Comparison of two groups with or without poor prognosis (mRS = 1) exhibited no significant difference in ICDSC scores. NS no significance. (C) There was a rapid and continent increase in the percentage of delirium patients (CAM‐ICU Score = 1) from 24 h of surgery. (D) The percentage of patients with delirium and high RASS scores rapidly increased. ***χ^2^ = 64.980, *p* < .0001.

The neural prognosis of postoperative pituitary adenoma patients was assessed by the modified Rankin scale (mRS) score at discharge, and all the enrolled subjects scored 0 or 1. Thus, we regarded patients with a mRS score = 1 as having a poor prognosis. A larger proportion of postoperative delirium patients had a poor prognosis at discharge than those without delirium (48.22% vs. 0.93%, χ^2^ = 48.762, *p* < .0001, Figure 2A). In addition, CAM‐ICU was used to determine the occurrence of delirium, while the ICDSC and RASS were used to evaluate the severity of delirium at five timepoints after surgery (i.e., within 6 h, 6–12 h, 12–24 h, 24–72 h, and after 72 h). Comparison of the highest ICDSC scores between the two groups with or without poor prognosis exhibited no significant difference (median ± IQR, 6.00 ± 2.00 vs. 7.00 ± 3.00, Z = −0.632, *p* = .528, Figure 2B). Finally, we found a rapid increase in the percentage of delirium patients (CAM‐ICU score = 1) and the proportion of patients with a high RASS score at 24 h after surgery during the hospital stay (χ^2^ = 64.980, *p* < .0001, Figure 2C and D). Univariate and multivariate analyses confirmed that delirium was an independent risk factor for poor neural prognosis in postoperative pituitary adenoma patients at discharge (OR 88.471, 95% CI 10.994–711.908, *p* < .0001, Table 3).

**TABLE 3:** Univariate and multivariate analysis: Variables associated with poor outcomes in pituitary adenoma patients.

	Univariate analysis					Multivariate analysis				
	*B*	*p*	OR	95% CI		*B*	*p*	OR	95% CI	
				Lower	Upper				Lower	Upper
Serum potassium ion at admission	–1.584	.033	0.205	0.048	0.880					
Serum sodium ion within 72 h of surgery	–0.011	.025	0.989	0.980	0.999					
Delirium	4.562	<.0001	95.737	12.021	762.479	4.483	<.0001	88.471	10.994	711.908

*Logistic regression* was used for univariate and multivariate analysis to establish a stepwise regression analysis model.

### 3.3 Hormonal and other blood risk factors for delirium in pituitary adenoma patients

Routine blood variables (i.e., album (ALB), serum chloride ion, serum potassium ion, serum sodium ion, glucose (GLU)) and hormonal variables (i.e., thyroid‐stimulating hormone (TSH), tetraiodothyronine (T_4_), corticotropin‐releasing hormone (CRH), insulin‐like growth factor 1 (IGF‐1), growth factor (GH), prolactin (PRL), follicle‐stimulating hormone (FSH), estradiol (E_2_)) were compared between the delirium and nondelirium groups. The results showed that compared to the delirium group, patients without delirium had a higher secretion of IGF within 72 h of surgery, as well as CRH after 72 h of surgery (116.55 ± 101.79 vs. 224.18 ± 312.31, *p* = .024; 13.87 ± 13.77 vs. 23.03 ± 18.74, *p* = .005). Conversely, the GLU level in the blood of the delirium group was higher than that in the nondelirium group 72 h after surgery (7.26 ± 2.79 vs. 5.64 ± 1.23, *p* = .023). The two‐group comparisons of other variables showed no significant difference (*p* > .05, Table 4).

**TABLE 4:** Comparison of hormonal and routine blood indicators between pituitary adenoma patients with or without delirium.

Variable	Occurrence of delirium		
	Yes	No	*p* Value
At admission			
ALB, g/L	42.08 (22.36)	39.79 (11.1)	.436
Serum chloride ion, mmol/L	16.83 (14.53)	20.63 (21.66)	.358
Serum potassium ion, mmol/L	3.91 (0.47)	5.59 (14.21)	.501
Serum sodium ion, mmol/L	105.27 (57.83)	116.4 (49.89)	.335
GLU, mmol/L	5.81 (1.98)	5.7 (1.3)	.760
TSH, μIU/mL	2.53 (3.19)	2.3 (1.89)	.624
T4, nmol/L	80.2 (35.52)	73.07 (40.61)	.375
CRH, pg/mL	26.58 (21.43)	29.24 (36.02)	.699
IGF, pg/mL	123.56 (109.45)	154.01 (172.29)	.406
Cortisol, nmol/L	346.97 (273.51)	374.37 (255.57)	.624
GH, ng/mL	0.8 (1.21)	4.06 (19.85)	.379
PRL, ng/mL	21.97 (33.67)	27.75 (80.03)	.703
FSH, U/L	16.59 (28.28)	16.81 (20.9)	.968
E2, pg/mL	24.45 (37.35)	28.69 (31.28)	.589
Testosterone, ng/mL	1.23 (1.67)	1.05 (1.42)	.598
Corporin, ng/mL	0.68 (0.53)	0.55 (0.71)	.492
Within 72 h of surgery			
ALB, g/L	37.32 (17.25)	35.14 (8.61)	.373
Serum chloride ion, mmol/L	48.31 (46.65)	36.33 (40.26)	.190
Serum potassium ion, mmol/L	4.22 (0.38)	4.14 (0.85)	.618
Serum sodium ion, mmol/L	121.43 (55.31)	136.83 (30.74)	.119
GLU, mmol/L	9.39 (4.85)	7.67 (7.88)	.379
TSH, μIU/mL	1.74 (1.94)	1.99 (9.33)	.875
T4, nmol/L	84.72 (44.13)	79.32 (45.63)	.546
CRH, pg/mL	54.95 (86.61)	41.76 (94.46)	.473
IGF, pg/mL	116.55 (101.79)	224.18 (312.31)	.024
Cortisol, nmol/L	683.21 (551.44)	677.15 (586.42)	.959
GH, ng/mL	0.9 (1.01)	1.59 (2.61)	.166
PRL, ng/mL	11.16 (14.67)	22.01 (101.57)	.645
FSH, mIU/mL	21.15 (36.28)	12.32 (14.82)	.387
E2, pg/mL	28.76 (18.49)	34.26 (62.8)	.766
Testosterone, ng/mL	3.19 (7.24)	2.99 (11.78)	.955
Corporin, ng/mL	1.89 (2.54)	1.57 (1.84)	.622
After 72 h of surgery			
ALB, g/L	35.71 (4.9)	33.67 (10.21)	.157
Serum chloride ion, mmol/L	38.66 (40.88)	34.38 (37.41)	.579
Serum potassium ion, mmol/L	4 (0.4)	5.53 (14.3)	.542
Serum sodium ion, mmol/L	132.25 (38.48)	122 (46.18)	.253
GLU, mmol/L	7.26 (2.79)	5.64 (1.23)	.023
TSH, μIU/mL	1.41 (2.06)	1.37 (1.54)	.899
T4, nmol/L	88.49 (49.56)	76 (39.83)	.149
CRH, pg/mL	13.87 (13.77)	23.03 (18.74)	.005
IGF, pg/mL	125.72 (144.88)	148.05 (234.54)	.643
Cortisol, nmol/L	424.01 (393.52)	427.61 (358.8)	.962
GH, ng/mL	0.47 (0.44)	0.9 (1.9)	.229
PRL, ng/mL	14.32 (38.88)	10.06 (9.63)	.452
FSH, mIU/mL	15.79 (25.29)	9.71 (12.28)	.417
E2, pg/mL	21.51 (23.98)	27.25 (31.02)	.461
Testosterone, ng/mL	1.87 (3.84)	0.72 (1.01)	.215
Corporin, ng/mL	0.63 (0.91)	1.3 (3.73)	.499

ALB, albumin; GLU, glucose; TSH, thyroid‐stimulating hormone; T_4_, tetraiodothyronine; CRH, corticotropin‐releasing hormone; IGF, insulin‐like growth factor; GH, growth factor; PRL, prolactin; FSH, follicle‐stimulating hormone; E_2_ estradiol.

Univariate analysis results indicated that serum potassium ions at admission (OR 0.311, 95% CI 0.103–0.935, *p* = .038), serum sodium ions within 72 h of surgery (OR 0.991, 95% CI 0.983–1.000, *p* = .048), GLU after 72 h of surgery (OR 1.655, 95% CI 1.180–2.320, *p* = .004), and CRH after 72 h of surgery (OR 0.964, 95% CI 0.936–0.994, *p* = .018) might be associated with the occurrence of delirium. Therefore, based on the above results, multivariate analysis confirmed that the elevation of GLU 72 h after surgery (OR 1.654, 95% CI 1.137–2.406, *p* = .008) independently increased the risk of delirium in postoperative pituitary adenoma patients (Table 5).

**TABLE 5:** Univariate and multivariate analysis: variables associated with delirium occurrence in pituitary tumor patients.

	Univariate analysis					Multivariate analysis				
	*B*	*p*	OR	95% CI		*B*	*p*	OR	95% CI	
				Lower	Upper				Lower	Upper
Serum potassium ion at admission	–1.169	.038	0.311	0.103	0.935					
Serum sodium ion within 72 h of surgery	–0.009	.048	0.991	0.983	1.000					
GLU after 72 h of surgery	0.504	.004	1.655	1.180	2.320	0.503	.008	1.654	1.137	2.406
CRH after 72 h of surgery	–0.036	.018	0.964	0.936	0.994					

*Logistic regression* was used for univariate and multivariate analysis to establish a stepwise regression analysis model.

GLU, glucose; CRH, corticotropin‐releasing hormone.

## 4 DISCUSSION

We investigated the potential perioperative risk factors for postoperative delirium in pituitary adenoma patients treated by endoscope‐assisted transsphenoidal surgery. We found that lower IGF‐1 and CRH levels were tightly associated with postdelirium and high levels of blood glucose (GLU) after surgery. Logistic regression analysis indicated that serum potassium and sodium levels and blood glucose levels in the perioperative period were independent risk factors for delirium.

Postoperative delirium is one of the most severe complications after surgery, with an acute confusion status characterized by inattention, cognitive dysfunction and an altered consciousness. It has been reported that delirium is an independent risk factor for the subsequent development of dementia (Richardson et al., 2021), and delirium in dementia patients can accelerate cognitive decline (Davis et al., 2017; Goldberg et al., 2020). Most patients in our cohort were approximately 50 years old and at high risk of developing cognitive impairment. Despite pituitary adenomas, high‐grade glioma was also reported to increase the risk of postoperative delirium, with insufficient compensation for injured brain regions involving cognition (Huang et al., 2022). Therefore, predicting, identifying and intervening to minimize the risk of delirium might reduce or prevent long‐term cognitive impairment (Fong & Inouye, 2022; Kong et al., 2022; Myles, 2020). In addition to the CAM‐ICU and ICHSC we used in this study (Gusmao‐Flores et al., 2012; von Hofen‐Hohloch et al., 2020), regional cerebral oxygen saturation may also be helpful for identifying the risk of delirium in postsurgery patients (Mutch et al., 2020; Susano et al., 2021).

Perioperative hydrocortisone has been widely used for pituitary adenoma patients to avoid postoperative hypopituitarism due to compression of the pituitary gland by the tumor and intraoperative injury to the pituitary (Higham et al., 2016; Husebye et al., 2021). Therefore, perioperative glucocorticoid supplementation has long been a routine practice for patients with pituitary adenomas (Hattori et al., 2021; Molitch, 2017). A recent randomized clinical trial indicated that withholding hydrocortisone was safe and noninferior to the conventional hydrocortisone supplementation regimen regarding the incidence of new‐onset adrenal insufficiency among patients with an intact hypothalamic‒pituitary‒adrenal axis undergoing pituitary adenomectomy (Guo et al., 2022). However, researchers did not take postoperative delirium into consideration in their study. Postoperative delirium is prevalent in patients after elective intracranial surgery and is associated with adverse outcomes and high cost (Wang et al., 2020). However, the association between hormone levels and delirium is still controversial. Only a few case reports have inspired us to pay close attention to hormone‐associated delirium (Burne et al., 2021; Li et al., 2017; Weng et al., 2008). In the present study, our data also indicate that lower IGF‐1 and CRH levels are tightly associated with postoperative delirium. Consistent with previous studies, low levels of IGF‐1 and high levels of GH are independently associated with the occurrence of delirium (Adamis et al., 2020; Li et al., 2018). Inversely, Chu et al. (2016) reported that serum IGF‐1 level was nonspecific for predicting POD onset, and IGF‐1 level alteration might be regarded as a disease biomarker rather than a risk marker. However, Chu et al. (2016) also noted that the results were influenced by factors such as the time of blood collection, basic medical disease, and sample size, which indicated that different study designs and data can lead to different conclusions. In this study, we used PSM to reduce the bias of age, gender and tumor size, attempting to provide more scientific evidence. In parallel, higher preoperative cortisol levels in cerebrospinal fluid are not associated with postoperative delirium in elderly hip fracture patients (Witlox et al., 2020). Compared to patients with nonfunctioning tumors, GH‐secreting tumor patients exhibit a higher incidence of sleep disturbance but not typical delirium symptoms (Kim et al., 2020). With growing evidence to support the withholding of hydrocortisone management for postoperative pituitary adenoma patients, normalized preventions, and interventions for delirium are needed in further studies and clinical practice (Scicutella, 2020).

To date, no pharmacologic drugs have been approved to specifically treat postoperative delirium (Scicutella, 2020). Haloperidol, dexmedetomidine, statin, and ketamine are not recommended for the prevention of delirium in all critically ill adults in current guidelines, and the evidence is low quality (Devlin et al., 2018). Recently, dexmedetomidine seems beneficial for attenuating neuroinflammation and likely to reduce the incidence of delirium, but it is limited to application in ICU practice and surgical patients (Swarbrick & Partridge, 2022), especially those who are mechanically ventilated (Moller et al., 2022). Intraoperative benzodiazepine restriction was reported to reduce the incidence of postoperative delirium in a recent large, multicenter, randomized trial (Spence et al., 2020). After endoscope‐assisted transsphenoidal pituitary adenoma resection, our data also indicated the importance of maintaining internal homeostasis. Serum potassium and sodium levels, as well as hyperglycemia, will increase the risk of postoperative delirium after surgery. According to several meta‐analyses, nonpharmacological multicomponent nursing and interventions may be effective in preventing and reducing the duration of delirium in ICU patients (Lange et al., 2022; Ludolph et al., 2020; Swarbrick & Partridge, 2022). Due to the lack of standard practice for nonpharmacological multicomponent interventions, more high‐quality evidence regarding the prediction, identification, and management of postoperative delirium is urgently needed.

At present, 95% of pituitary adenoma patients are treated by endoscope‐assisted transsphenoidal surgery, which introduces much less iatrogenic damage than transfrontal craniotomy or radiotherapy (Goudakos et al., 2011; Kanat et al., 2022; Yadav et al., 2012). Thus, postoperative cognitive impairment should not be aggravated in theory. Wang et al. (2017) compared traditional surgery and endoscope‐assisted transsphenoidal surgery and reported that difference in the surgery approaches did not have a significant effect on postoperative cognitive changes. Controversially, Leistner et al. (2015) considered physical suffering after endoscope‐assisted transsphenoidal surgery, such as rhinogenic headache, deep cavity nasal discomfort, and even negative emotions, as potential inducements for POD. It is still hard to affirm whether endoscope‐assisted transsphenoidal surgery contributes to the occurrence of POD. Regrettably, we did not enroll patients who had undergone other surgery approaches, which needs to be further studied in the future.

## 5 CONCLUSION

In summary, our study indicated that lower IGF‐1 and CRH levels, as well as serum potassium and sodium levels and blood glucose levels, were potentially associated with postoperative delirium in pituitary adenoma patients treated by endoscope‐assisted transsphenoidal surgery. These data might provide preliminary evidence for the management of postoperative delirium in pituitary adenoma patients after surgery, but further studies on pharmacological and nonpharmacological multicomponent interventions are needed.

## AUTHOR CONTRIBUTIONS

**Jin Liu**: Conceptualization, methodology, formal analysis, investigation, data curation, writing—original draft. **Jinyu Qian**: Conceptualization, methodology, investigation, data curation, writing—original draft. **Xia Wang**: Investigation, data curation. **Jie Lin**: Methodology, formal analysis, data curation, writing—original draft, visualization. **Sunyan Yang**: Investigation. **Rong Hu**: Investigation, resources, supervision. **Jishu Xian**: Investigation, resources, supervision. **Hua Feng**: Resources, supervision, funding acquisition. **Yujie Chen**: Conceptualization, methodology, writing—review & editing, supervision, funding acquisition. **Binbin Tan**: Conceptualization, methodology, writing—review & editing, supervision, project administration, funding acquisition. All authors read and approved the current version of manuscript.

## CONFLICT OF INTEREST STATEMENT

The authors declare that they have no competing interests.

### PATIENT CONSENT STATEMENT FOR WORK WITH HUMAN SUBJECTS

Informed consent to participate was waived by the Ethics Committee of Southwest Hospital of Army Medical University due to the observational nature of the study.

### CLINICAL TRIAL REGISTRATION

The present study was registered in the Chinese Clinical Trial Registry (http://www.chictr.org.cn/enIndex.aspx, Trial No. ChiCTR2100053607, Registration Date: November 25, 2021).

### PEER REVIEW

The peer review history for this article is available at https://publons.com/publon/10.1002/brb3.3041.

## Supporting information

Supplemental Figure S1. Comparison of the standard mean difference of baseline data before and after propensity scoring matching.

## Data Availability

The data that support the findings of this study are available on request from the corresponding author. The data are not publicly available due to privacy or ethical restrictions.
