# Supplementary material for: Identifying hormones and other perioperative risk factors for postoperative delirium after endoscope‐assisted transsphenoidal pituitary adenoma resection: A retrospective, matched cohort study
Source: Brain Behav. 2023 May 3;13(7):e3041. doi: 10.1002/brb3.3041 (PMC10338746; doi:10.1002/brb3.3041)
Supplement: Supplementary file 1 — Supplemental Figure S1. Comparison of the standard mean difference of baseline data before and after propensity scoring matching. [file BRB3-13-e3041-s001.docx]

**Supplemental Materials**


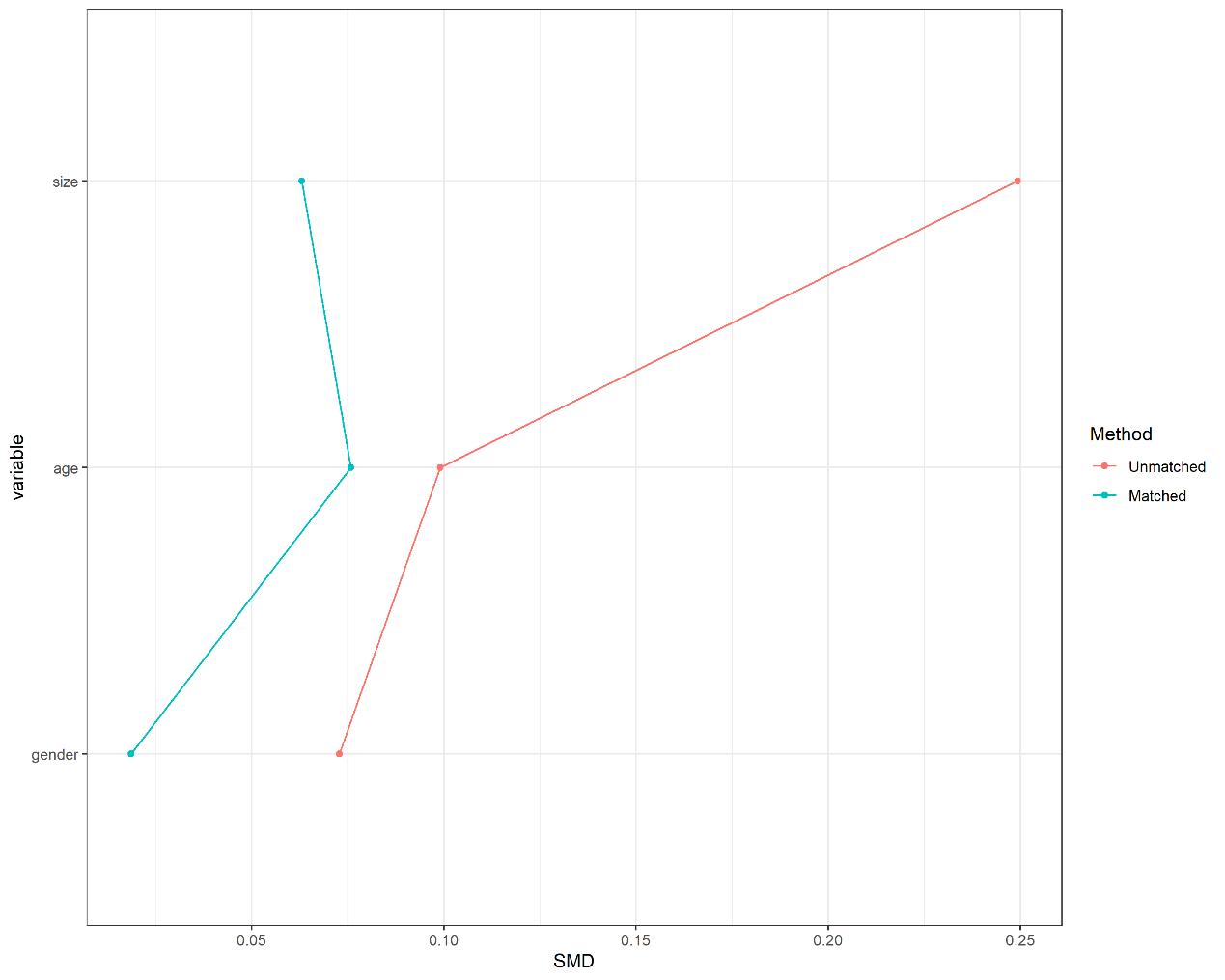


**Supplemental Figure 1. Comparison of the standard mean difference of baseline data before and after propensity scoring matching.**

The black line represents the SMD value equal to 0.1. The SMD values of confounder factors, including age, sex, and tumor size, were all less than 0.1 after propensity score matching, namely, the differences between the two groups could be ignored. SMD Standard Mean Difference
